# Barriers and facilitators to growth monitoring and promotion in Nepal: Household, health worker and female community health volunteer perceptions

**DOI:** 10.1111/mcn.12999

**Published:** 2020-07-12

**Authors:** Madeline M. Pollifrone, Kenda Cunningham, Pooja Pandey Rana, Morgan M. Philbin, Shraddha Manandhar, Krishna P. Lamsal, Raj Nandan Mandal, Vikash Deuja

**Affiliations:** ^1^ Department of Sociomedical Sciences Columbia University Mailman School of Public Health New York New York USA; ^2^ Helen Keller International Lalitpur Nepal; ^3^ Nepali Technical Assistance Group Kathmandu Nepal; ^4^ FHI 360 Kathmandu Nepal

**Keywords:** child nutrition, community health workers, growth, growth monitoring, health facilities, Nepal

## Abstract

Growth monitoring and promotion (GMP) is both a service for diagnosing inadequate child growth in its earliest stages and a delivery platform for nutrition counselling. The widespread use of GMP services in developing countries has the potential to substantially reduce persistent child undernutrition through early diagnosis and by linking caregivers and their children to key health and nutrition services. However, researchers have questioned the effectiveness of GMP services, which are frequently undermined by underdeveloped health systems and inconsistent implementation. This analysis examined both supply‐ and demand‐side factors for GMP utility in Nepal from the perspectives of beneficiaries and service providers, particularly focusing on three components of GMP: growth assessment, analysis of growth status and counselling. The most common factors influencing GMP uptake included beneficiaries' perceptions of the relative importance of GMP and the knowledge and skill of frontline workers. Both providers and beneficiaries viewed GMP as a secondary health and nutrition activity and therefore less important than curative services. We found deficits in GMP‐related knowledge and skills among providers (i.e. health workers and female community health volunteers), as well as indications of poor training quality and coverage. Furthermore, we found variation in GMP utilization by maternal age, education and residency (alone, nuclear or extended), as well as household socio‐economic well‐being and rurality. This study is the first to assess factors influencing both beneficiaries and service providers for GMP utilization. Further research is needed to explore the implementation of improved GMP protocols and to evaluate facility‐level implementation barriers.

**Key messages**

- Growth monitoring and promotion (GMP) is a preventative and promotive nutrition activity.
- Globally, there is limited evidence on the factors that affect the implementation and utilization of GMP from the sides of both providers and beneficiaries.
- In Nepal, GMP has the potential to alter the landscape of child undernutrition if systematic changes to country‐level protocols are researched, designed and implemented.

## 1 INTRODUCTION

Nearly half of all deaths worldwide among children under 5 years of age are linked to undernutrition (Black et al., 2013). Nutritional well‐being during this critical period has both immediate and lasting consequences on a child's physical and cognitive health, development and functioning (English, Peer, Honikman, Tugendhaft, & Hofman, 2017; Hossain et al., 2017; Martorell & Woodruff, 2017; Pietrobelli et al., 2017). To combat child undernutrition, many interventions target households in the 1,000‐day period from gestation to the child's second birthday (Martorell & Woodruff, 2017; Schwarzenberg & Georgieff, 2018). Although interventions that support optimal nutrition beyond the first 2 years of life are important, damage sustained during the first 1,000 days is often irreversible (World Health Organization [WHO], 2013).

Interventions that target early diagnosis and corrective action for child undernutrition have been prioritized globally, particularly in low‐ and middle‐income countries (LMICs) (Ashworth, Shrimpton, & Jamil, 2008). Growth monitoring and promotion (GMP), for example, is used in many LMICs to diagnose inadequate child growth in its earliest stages and in turn alter the child's growth trajectory through nutrition counselling and other health‐promoting actions. According to WHO guidelines, GMP includes (1) the routine measurement of a child's weight and length/height; (2) the plotting of the child's measurements and comparison of the child's status to a standardized growth chart to assess growth adequacy; (3) growth‐informed counselling; and, if necessary, (4) the undertaking of remedial, health‐promoting action (WHO, 2006, 2008).

When implemented correctly, GMP programmes have created linkages to key preventative and curative health services, increased mothers' knowledge of proper infant and young child feeding practices and provided the opportunity for early diagnosis and treatment of undernutrition (Adhikari, Khatri, Paudel, & Poudyal, 2017; Ashworth et al., 2008; Gyampoh, Otoo, & Aryeetey, 2014). Before the 1990s, GMP services were the subject of much enthusiasm, research and evaluation, but the attribution of GMP service utilization to positive changes in a child's growth status has long been the subject of debate (Ashworth et al., 2008; Garner, Panpanich, Logan, & Davies, 2000). Although GMP services are still in use in most LMICs, its efficacy is limited by numerous challenges including low service coverage, inadequate training of health workers and resulting measurement errors, incorrect interpretation of growth charts, and poor or nonexistent counselling (Ashworth et al., 2008; Bégin et al., 2019; de Onis et al., 2012; Feleke, Adole, & Bezabih, 2017; Laar, Marquis, Lartey, & Gray‐Donald, 2018; WHO, 2006; WHO & UNICEF, 2009). In Nepal, where the prevalence of underweight (27%), stunting (36%) and wasting (10%) remains high despite incredible progress over the last 20 years, GMP is a prioritized nutrition intervention (Cunningham, Headey, Singh, Karmacharya, & Rana, 2017; Ministry of Health, 2017). Nepal's Multi‐Sector Nutrition Plan‐II (MSNP‐II) (2018–2022) aims to address the complex causes of malnutrition by scaling up both nutrition‐specific and nutrition‐sensitive services and improving utilization of these services (Government of Nepal National Planning Commission [NPC], 2017). Nepal's MSNP‐II frames GMP as a key platform for improving infant and young child nutrition and care and recommends that children under 2 years of age receive monthly GMP (NPC, 2017). Health workers are responsible for conducting GMP services—which focus on weight for age, not height—at local health facilities or at monthly primary health care outreach clinics (Child Health Division, 2016; de Onis et al., 2012). A cadre of more than 52,000 female community health volunteers (FCHVs), who serve as the first point of contact in communities across Nepal and refer people into the health system, support the implementation of GMP services (Ministry of Health and Population, 2019).

In this paper, we use quantitative and qualitative data to assess the first three of four components of GMP services—routine anthropometric measurements to assess growth, plotting and comparing child growth, and growth‐informed promotive counselling—as noted by the WHO definition above. Specifically, for each of these three stages, we examine the current state of GMP service provision and utilization in Nepal and identify barriers and facilitators to optimal GMP service from both service provider and beneficiary perspectives. We used Andersen's Behavioral Model of Health Service Use as a framework for our exploration of both contextual and individual factors that predispose and enable GMP service success in Nepal (Andersen, 1968, 1995, 2008). We hypothesize that routine GMP service use is minimal and that there are both individual and structural health system barriers contributing to gaps in coverage and delivery of high‐quality GMP services. These analyses will help to fill not only gaps in research related to health services in Nepal but also global research gaps related to GMP failures and opportunities for improvement from both beneficiary and provider perspectives.

## 2 METHODS

### 2.1 Quantitative data collection and management

The quantitative data used are from a cross‐sectional monitoring survey of *Suaahara II*, a United States Agency for International Development (USAID)‐funded, multisectoral nutrition programme that aims to improve the health and nutrition status of mothers and children in 42 of Nepal's 77 districts. New ERA, a local survey firm, collected data from June 10 to September 10, 2017. Multistage cluster sampling and probability proportion to size (PPS) techniques were used to select the following: *Suaahara II* districts (*n* = 16), one rural and one urban municipality per district (*n* = 32), three wards per municipality (*n* = 96) and two clusters per ward (*n* = 192). For the final stage, 19 households with a child under 5 years were randomly selected from each cluster from a full list gathered by the survey firm (*n* = 3,648) (Suaahara II, 2018). The household survey collected key information on a variety of indicators, including household socio‐economic and demographic characteristics, nutrition‐ and health‐related knowledge and practices, and utilization of Government of Nepal health and nutrition services. Additionally, one FCHV from each cluster (*n* = 192) and one health facility key informant from each ward (*n* = 96) were included in the survey. The FCHV and health facility key informant (preference for those in the highest level role of health facility in‐charge, when available) questionnaires gathered data on socio‐economic and demographic characteristics; exposure to training on key health and nutrition areas; perceptions of their work experience; exposure to *Suaahara II* platforms; and their detailed knowledge and skills related to counselling and following government protocols, including GMP (Suaahara II, 2018).

All data were collected electronically using Open Data Kit software on Android phones. Once collected and reviewed by a supervisor, the data were synced to a secure server. New ERA staff checked the quality and consistency of data and completed the first round of data cleaning and verification, as well as the translation of open‐ended responses into English when necessary. *Suaahara II* staff further cleaned the data, including variable generation. The de‐identified and cleaned data files were then used for this analysis. Of the 3,648 households surveyed, about half (*n* = 1,850) had a child under 2 years of age. These households were the focus of this study. Of the 96 health facilities surveyed, this analysis focuses only on the health posts (*n* = 91); two hospitals and three primary health care centres, which have different mandates and scopes of practice than health posts, were excluded to avoid the introduction of extreme heterogeneity.

### 2.2 Qualitative data collection and management

In July 2018, a qualitative study was done to complement the prior quantitative survey's descriptive findings and enable deeper exploration of barriers and facilitators for participation in GMP services. Data were collected from mothers, frontline workers (health facility workers and FCHVs), and *Suaahara II* national and district staff by Square One, a local survey firm. A purposive sampling strategy was used to select districts representative of Nepal's three agroecological zones (i.e. Terai, hills and mountains)—Rupandehi, Bhojpur and Bajhang—as well as individuals with relevant knowledge and experience at national, district and community levels. There were 37 data collection points split equally across the three districts; data were collected through focus group discussions (FGDs) with *Suaahara II* staff, health workers and FCHVs, and 1,000‐day mothers (one FGD with *Suaahara II* staff, one per district with health workers and one per district with mothers; *n* = 7) and in‐depth interviews (IDIs) with 1,000‐day mothers (10 IDIs per district; *n* = 30). During the FGDs, which ranged from five to 13 participants, one researcher facilitated the discussion while another researcher took notes. While most FGDs were conducted in Nepali, interviews were conducted in Awadhi and Bhojpuri in Rupandehi and similarly, Doteli language was used for interviews in Bajhang.

All qualitative data were digitally recorded, transcribed verbatim and translated to English from Nepali, Awadhi, Bhojpuri and Doteli by the local survey team. De‐identified IDI and FGD transcripts were uploaded into Atlas.ti 8.2 for data management and analysis.

### 2.3 Analyses

We used the quantitative data to summarize survey respondents' background information and GMP service uptake in the survey population. Potentially predisposing factors, such as mother's age, education, caste/ethnic group, religion, occupation, agroecological zone, residence, child age and child sex were explored at the bivariate level (Table 1). Age and level of education were constructed as continuous variables with education level referring to the total number of years of formal schooling received. Caste/ethnicity was categorized into three groups: socially advantaged (Brahmins/Chhetri), socially excluded (Dalit, Muslim and disadvantaged Janajati) and other groups (Gurung/Thakali, Newar, other non‐Dalit Terai castes and others) (Aasland & Haug, 2011; Pandey, Dhakal, Karki, Poudel, & Pradhan, 2013). Household socio‐economic status was measured using the Equity Tool, which generates quintiles based on ownership of key assets and quality of household structures (Metrics for Management, 2015). On the provider side, we summarized frontline workers' background information, formal training, and knowledge and skills related to GMP to describe the services available and contextualize the health care environment in which care is sought and provided (Table 2). All analyses were conducted using Stata/IC 15.1 software.

**TABLE 1:** Maternal characteristics and utilization of growth monitoring and promotion services

Sample characteristics	All (*N* = 1850)	Ever received GMP (*N* = 1,652)	Never received GMP (*N* = 198)	Significance of differences: *p* value
	Mean (SD)/%			
		Mean (SD)/%	Mean (SD)/%	
Age (in completed years; range: 15–49)	24.9 (5.2)	24.8 (5.1)	26.3 (5.7)	<0.001
Education (in completed years of formal schooling; range: 0–18)	6.6 (4.2)	6.9 (4.1)	4.2 (4.2)	<0.001
Main occupation: agriculture	58.4%	58.2%	59.6%	0.891
Household size (range: 2–34)	5.3 (2.4)	5.2 (2.3)	5.6 (3.0)	0.029
Maternal residency				0.002
Alone with children	15.9%	15.4%	20.2%	
Husband (and children) only	27.7%	26.9%	34.9%	
Maternal family	2.4%	2.5%	1.0%	
Paternal family	54.0%	55.2%	43.9%	
Other	0.1%	0.1%	0.0%	
Child age (in completed months; range: 0–23)	11.4 (6.9)	11.3 (6.8)	11.7 (7.6)	0.447
Child sex: female	46.0%	45.4%	50.5%	0.174
Caste/ethnicity				0.093
Brahmin/Chhetri	40.0%	41.3%	29.3%	
Socially excluded	48.5%	48.3%	50.0%	
Other	11.5%	10.4%	20.7%	
Socio‐economic well‐being				0.001
Equity Quintile 1 (lowest)	21.0%	19.1%	36.4%	
Equity Quintile 2	28.5%	29.5%	20.7%	
Equity Quintile 3	23.2%	24.0%	16.7%	
Equity Quintile 4	21.0%	20.8%	22.7%	
Equity Quintile 5 (highest)	6.2%	6.5%	3.5%	
Decision‐making power: child health care				0.294
Little to no input	0.8%	0.9%	0.5%	
Input into some decisions	10.1%	9.7%	13.1%	
Input into most or all decisions	88.9%	89.2%	86.7%	
No decisions made	0.2%	0.2%	0.0%	
Agroecological zone				0.077
Terai	32.1%	31.7%	35.4%	
Hills	55.0%	56.7%	40.9%	
Mountains	12.9%	11.6%	23.7%	
Residence: rural area	49.5%	51.6%	32.3%	<0.001
GMP utilization				
Received GMP (ever)	89.3%	—	—	
Time of the last GMP by health professional, among those who had ever used				
Within the last 3 months	—	37.2%	—	
Within the last 6 months	—	75.4%	—	
Within the last 9 months	—	85.9%	—	
Within the last 12 months	—	90.7%	—	

Abbreviation: GMP, growth monitoring and promotion.

**TABLE 2:** Health facility worker and FCHV characteristics

Sample characteristics	Health facility workers (*N* = 91)	FCHVs (*N* = 192)
	Mean (SD)/% (*n*)
		Mean (SD)/% (*n*)
Age (in completed years; range: 18–70, 20–58)	34.3 (9.8)	41.1 (11.0)
Experience (in completed years; range: 0–29, 0–31)	10.5 (9.3)	15.8^a^ (8.8)
Education (in completed years of formal schooling; range: 0–16)	—	5.7 (4.2)
Literacy: can read whole sentence	—	78.1%
Respondent sex: female	15.4%	100.0%
Health post position		
Medical officer	5.5%	—
SN/HA	60.4%	—
Sr AHW/AHW	30.7%	—
Sr ANM/ANM	2.2%	—
Administrative staff	0.0%	—
Other	1.1%	—
Caste/ethnicity		
Brahmin/Chhetri	65.9%	55.7%
Socially excluded	12.1%	30.2%
Other	22.0%	14.1%
Agroecological zone		
Terai	31.9%	31.3%
Hills	56.0%	56.3%
Mountains	12.1%	12.5%
Residence: rural area	52.7%	50.0%
Training received		
Measuring weight of children <2	76.9%	56.3%
Adequacy/quality of child's diet	73.6%	88.0%
Counselling methods	47.3%	35.4%
Level of agreement: received adequate training to meet current responsibilities		
Strongly disagree	3.3%	5.7%
Disagree	31.9%	18.8%
Neither agree nor disagree	3.3%	7.3%
Agree	40.7%	47.9%
Strongly agree	20.9%	20.3%
Number of days per month GMP is provided at health facility (range: 1–30)	19.0	—
Conducted growth monitoring (measuring weight) in the last month	—	50.0%
Classifying nutritional status of child <5 years		
Correct reading (improving but still in red [concern] area)	34.1%	21.4%
Incorrect reading	65.9%	56.8%
Did not know	0.0%	21.9%
Knowledge of steps to diagnose growth faltering in children <2 years		
Named all 5 steps	8.8%	0.5%
(1) Weigh child as per protocol	79.1%	56.3%
(2) Record weight in graph in child health card	48.4%	13.5%
(3) Draw line to connect weight taken in different months	40.7%	16.2%
(4) Match with curve shown in child health card	46.2%	22.0%
(5) Identify the growth trend (inclining, stagnant or declining)	46.2%	17.7%
Could not recall name any step	0.0%	29.2%

Abbreviations: AHW, assistant health worker; ANW, auxiliary nurse midwife; FCHV, female community health volunteer; GMP, growth monitoring and promotion; HA, health assistant; SN, staff nurse.

a Among those who remember (*N* = 190).

The qualitative data were analysed using a content analysis approach to identify barriers to GMP service utilization and provision as well as potential solutions at the beneficiary and service provider levels. The transcripts were read repeatedly as part of the data familiarization process and then hand coded by the lead author who created a preliminary codebook containing key concepts and categories after reading a cross section of the interviews. Thematic codes from existing literature were identified and integrated into the codebook to ensure that both theory‐based and emergent concepts were included. The lead author then applied these codes to all interviews to consolidate and create more nuanced versions of the codes. These codes were used to compare responses across data points and were then gathered into several conceptual categories. Finally, selective coding was completed to generate results. The co‐authors held regular meetings with the lead author to ensure that any questions or potential discrepancies were addressed. The lead author also consulted with Square One Research and Training, the research company who conducted the interviews, to ensure that she had correctly interpreted the transcripts.

### 2.4 Ethical considerations

Ethical approval from the Nepal Health Research Council was received for both quantitative and qualitative studies. Participation in the study was voluntary, and written informed consent was obtained from each respondent prior to beginning each questionnaire and interview.

All data collection was approved by the Nepal Health Research Council. Written informed consent was obtained from each quantitative survey respondent prior to beginning any interview, and verbal consent to continue the survey was obtained after the completion of each section in the questionnaire. Similarly, for the interviews and FGDs, written informed consent was obtained prior to any data collection (Suaahara II, 2019).

## 3 RESULTS

### 3.1 Sample characteristics

The majority (60.0%) of surveyed households with children under 2 years belonged to a socially excluded caste/ethnic group (Table 1). Among all mothers, 58.3% reported their primary occupation as agriculture or livestock farming, whereas another 33.3% reported a nonearning position such as housework. The majority (54.0%) of mothers lived with their husband's family, and the average household size was 5.3 inhabitants (range: 2–34 inhabitants). On average, mothers were 24 years old (range: 15–49 years) and children were 11 months old (range: 0–23 months). A slight majority (54.1%) of the children under 2 years were male.

Among the health facility informants surveyed, the majority (60.4%) held the higher level position of nurse/health assistant, per design (Table 2). On average, they were 34 years old (range: 20–58 years) and had 11 years of experience (range: 0–32 years). The health facility workers interviewed were predominantly male (84.6%) and belonged to the high caste groups Brahmin or Chhetri (65.9%). More than half (55.7%) of FCHVs in the survey sample (*n* = 192) were also Brahmin/Chhetri. FCHVs were on average 41 years old (range: 18–70 years) with an average of 16 years of experience (range: 0–30 years). More than three of four FCHVs were literate (78.1%).

### 3.2 Routine anthropometric measurement to assess growth

Nearly 90% of mothers with children under 2 reported having ever used GMP services (Table 1). There was no significant difference in GMP use by child sex (90.2% vs. 88.2% for male and female children) or age (11.3 months for those that ever received vs. 11.7 months for those who did not). Although nearly all reported that they had attended GMP at some point, only slightly more than one third of those who had ever attended had been to GMP in the 3 months prior to the survey. On the provider side, health workers reported that GMP provided an average of 19 days per month at their post (range: 1–30) (Table 2). Exactly half (50.0%) of FCHVs reported that they had conducted growth monitoring (i.e. measuring the weight of children) in the last month.

The most frequently recurring theme across interviews with both providers and beneficiaries was the limitations on frontline workers' capacity to provide quality, comprehensive care through GMP services. Discrepancies in training for frontline workers were frequently cited as a driver of suboptimal GMP. Frontline worker training was done primarily through government supervisors, but training on specific topics such as GMP was also implemented by external development partners, including *Suaahara II.* When asked about GMP protocol, FCHVs in the focus group in Bajhang noted that all FCHVs had received government training on GMP in recent months and that others ‘should not have forgotten about it within a year’. However, only 76.9% of health facility workers and 56.3% of FCHVs reported that they had received government training on the proper measurement of a child's weight. Overall, 35.2% of health facility workers and 24.5% of FCHVs did not feel as though they had received adequate training to meet their current responsibilities. A *Suaahara II* staff member in Bajhang (FGD) explained that training and capacity building for all frontline workers should be prioritized:

For now, no support exists to teach (health facility workers) how they should be recording measurements in their registers …. When I ask representatives of other districts, ‘Have you taught anyone how to fill out the registers?’ they tell me that they have not. In fact, they say that they do not know how to do it themselves. While we talk about the quality of work, we have not focused much on how we are going to build the capacity of our staff. This could mean providing them with training.

Furthermore, we identified three major issues related to the provision and utilization of GMP services, particularly the routine measurement of a child: (1) distance and sociocultural constraints for caregivers to take children to GMP; (2) preference given to immunization and other curative services over GMP by providers and beneficiaries alike; and (3) discrepancies around FCHVs' role in GMP services in policy versus practice.

First, as Nepal is a predominantly rural country with limited road networks, distance was a major barrier that often deterred or prevented mothers from accessing GMP services. In Bhojpur and Bajhang, hilly and mountainous terrain added significant strain to mothers' commutes. Mothers recounted the challenges associated with carrying their child long distances for health services, particularly as the child aged. In one interview, a young mother in her early 20s (Bhojpur; IDI) gestured to her 16‐month‐old son and told the interviewer:

Now, look at this child. I have to carry him, whether on my back or in my arms. Is it possible to walk while carrying a child of this size? It is very difficult. If the road was flat, then it would have been easier, but it's uphill.

In the same district, mothers said that attending the clinic would be easier if roads and transportation were more accessible. Distance and transportation challenges were often compounded by seasonality and extreme weather. Referring to flooding, landslides and washed‐out roads, one mother in her mid‐20s (Bajhang; IDI) frankly stated, ‘Everything is difficult when it rains’.

Furthermore, the demands of long hours of agricultural and domestic work also prevented caregivers from attending GMP services, as many did not have the ability to attend. Because of gendered divisions of labour, mothers were primarily responsible for seeking care for the child, regardless of their workload. This point was echoed by mothers in their mid‐20s in a focus group in Bhojpur:

Interviewer Do the mothers have to go there [to GMP at the health facility] to weigh the child or can someone else take [the child]?

Participant 1 Others can take the child as well.

I But that has not happened? What about fathers?

P1 He can bring them, but he does not obey.

I He does not obey?

Participant 4 He needs to take it [the child].

Participant 3 He says, ‘You take it’. He gets lazy.

P4 (Laughs) Too lazy to carry his child.

P1 He says, ‘If there is no work to do, then take it. But if you need to cut grass [a common household chore done to gather feed for oxen] then why does it need to be weighed?’ And that is how it is.

In Nepal, young wives traditionally live with their husband's families, and as a result, in‐laws often have great influence over childcare. Although only mothers in Rupandehi reported that their mobility was explicitly restricted by their husbands or in‐laws, mothers in all districts noted that their movement (e.g. trips to the health post, market and community space) was often questioned by other household members. According to mothers from Bhojpur:

Participant 3 If I tell [my in‐laws] I want to go places then my father‐in‐law says, ‘Why do you need to go?’ Things like that.

Participant 1 ‘Since you know everything, nothing will come from asking me, house ox [a disapproving nickname]’. (Everyone laughs.)

P3 I cannot win no matter what I do.

P1 We cannot win over our mother‐in‐law and father‐in‐law no matter what.

Second, it was reported that GMP was often being administered at the Expanded Programme on Immunization outreach clinics led by local health facilities. As *Suaahara II* staff (Rupandehi district staff; FGD) noted, ‘The human resource is focused on vaccination while there is lack of manpower regarding GMP, even though the weighing machine is available’. In all three districts, mothers placed a greater emphasis on technical or curative services—such as vaccination or treatment for a sick child—than on pursuing GMP on its own. Because GMP was usually offered at the time of the local health facility's immunization outreach clinics, some mothers suspended their utilization of GMP services when their child's vaccination schedule was completed, despite knowing that GMP should be conducted monthly until their child reached 2 years of age. When asked why she had not taken her child for GMP in 3 months, one young mother (Bhojpur; IDI) laughed and said, ‘Now, it is what it is—because I don't have to get vaccinations for my child anymore, I have lost interest’. When asked why her child was not weighed the previous month, another mother in her early 20s (Bhojpur; FGD) explained, ‘I didn't go up there [to the health facility] for vaccination last month. I went to get vaccinated but turned back half‐way because I was told the vaccines were out’. Many mothers did not see the significance in attending GMP services when a child was not visibly ill, as GMP services alone did not warrant a trip to the local health facility. An ill child would be weighed and measured at a health facility but only to collect key health indicators—not for monitoring growth over time with the intention of counselling to ensure proper growth in the future. As one *Suaahara II* staff member (Kathmandu staff member; FGD) summarized:

So how will they be motivated to go there regularly? For example, when they go there if the child has diarrhea, they get zinc and ORS [oral rehydration salts]. But they think, ‘What could I possibly get if I go for growth monitoring?’ There needs to be a change in people's perceptions and they should realize that it is for their own good. They should understand that the benefits are not instant and that it will take some time.

Third, although Nepal's GMP strategy suggests that FCHVs should simply support, not conduct, GMP services, we found that implementation often differed from protocol. There were frequent suggestions to engage FCHVs as providers of GMP services. As one mother in her early 20s (Rupandehi; IDI) explained, ‘If they [FCHVs] could be trained to conduct GMP and come door‐to‐door then it will be easier for us to participate in GMP’. In the FGD in Bajhang, another mother noted that GMP is now done by FCHVs in her monthly Health Mothers' Group (HMG) meetings:

In our HMG meeting, they [FCHVs] always take the measurements. There is a weighing machine as well …. It wasn't there before, but from this year onwards, they have been measuring the weight of our children. The FCHV even asks us to bring our children to the meeting.

### 3.3 Plotting and comparing child growth

Both health facility workers' and FCHVs' knowledge and skills related to GMP were assessed by presenting them with a hypothetical child's growth chart in which the child's measurements were in the ‘red’ area of the chart with a positive growth trend, indicating that the child had severe acute malnutrition but was improving. When presented with this sample chart, 65.9% of health facility workers and 78.6% of FCHVs incorrectly classified the child's status or did not know how to classify the status (Table 2). Furthermore, only 8.8% of health workers and less than 1.0% of FCHVs were able to name all five steps used in the process of diagnosing growth faltering among children under 2 years of age: (1) weigh the child following protocol; (2) record the weight in the growth chart in the child's health card; (3) draw a line connecting the weight taken across several months; (4) match the line with the curve indicating the child's health status (green, yellow and red), as shown in the child health card; and (5) correctly identify the growth trend (improving, stagnant and declining).

In all three districts, there were reports of frontline workers failing to record the child's measurements, which prevents monitoring of the child's growth trend. In some cases, mothers took it upon themselves to memorize their child's measurements, knowing that the measurements were not being recorded by frontline workers. As a *Suaahara II* district staff member from Bhojpur (FGD) summarized:

The child's measurements are not noted in the child health card [by the frontline workers] … I have seen this when I have gone for a visit to primary health care outreach clinics. They do not note it down on the graph. And in that case, we cannot track whether the child's growth is good, if it's constant, or if it's degrading.

Even when measurements were recorded and plotted on a growth chart, issues arose regarding the interpretation of a child's growth.

### 3.4 Growth‐informed counselling

Our survey results showed that among caregivers who had ever taken their child to GMP, 70.9% were not told about their child's growth (i.e. the change in the child's weight over time) during their last GMP (Table 1). Furthermore, among mothers whose child had ever received GMP, only 21.0% reported discussing child nutrition and 12.7% reported discussing child health with an implementing health frontline worker during their last GMP. Although most frontline workers (76.9% of health facility workers and 88.0% of FCHVs) had received training on assessing the adequacy of a child's diet, less than half received training on counselling methods (47.3% of health facility workers and 35.4% of FCHVs) (Table 2).

A *Suaahara II* staff member from Bhojpur (FGD) expressed frustrations with the insufficiency of GMP services in health facilities, particularly related to using the data for nutrition counselling:

We taught them [health facility workers] how to fill the growth charts out and how to draw the line. We also told them that they should provide counseling and that it's not enough just to draw the line. It would not be considered growth monitoring and promotion if only the lines are drawn; counseling is also needed.

In all three districts, understaffing of government health facilities was a significant barrier to the delivery of quality care, including less comprehensive counselling per patient.

The major thing is time …. Sometimes they have an overload of work while other times the mothers do not have the patience to wait for 5 minutes and listen to what the health workers have to say. (Kathmandu staff member; FGD)

Most mothers, however, felt positively about their interactions with health facility workers and FCHVs throughout the GMP process. When the interviewer asked what she liked most about the GMP services, one mother in her early 40s (Rupandehi; IDI) responded by detailing her conversations with an FCHV about child feeding practices:

Everything was good. I like the way they speak to the mothers the most. The Female Community Health Volunteers were speaking politely with all mothers. I also like the way they were suggesting nutritional food that we must provide to our baby. They have also suggested that we eat food more regularly and give food regularly to our baby, as well.

Another mother in her mid‐20s shared similar positive experiences (Bajhang, IDI):

They [frontline workers] treat us very well. If their behavior is not good, why would we go there? They speak to us like we are familiar with each other. When people have good behavior and speak politely, well … just see how we have gotten along in just a day! I feel like you are my own family member (laughs). That is how they make me feel, too.

## 4 DISCUSSION AND CONCLUSION

Comprehensive GMP services consist of several steps, including the weighing and measuring of a child, recording of measurements and interpretation of the growth chart, and provision of informed, nutrition‐promotive counselling. Using both quantitative and qualitative data, we assessed these three components of GMP services, which each require skilled health service providers. For each stage, we identified both provider and beneficiary perspectives on the major barriers and facilitators to optimal GMP services in Nepal. We found that although awareness of GMP was high, routine utilization was low and both demand and supply‐side constraints existed. Our findings were framed by Andersen's Behavioral Model of Health Service Use, which highlights the influence of a combination of household‐level and health system‐level factors on health service utilization. Our findings indicate that perception (i.e. health belief) of the relative importance of GMP versus other services served as a critical predisposing factor to GMP utilization. Both mothers and providers in the study had overwhelmingly neutral attitudes towards routine GMP, despite high levels of service availability, awareness and reported utilization. Although knowledge regarding the importance of GMP is widespread, both providers and beneficiaries indicated that the service is largely secondary to competing priorities, including utilization of other health services with more visible technical and curative components such as vaccination or the treatment of child illness. This finding was consistent with several studies in other LMICs (Roberfroid, Lefèvre, Hoerée, & Kolsteren, 2005; Feleke et al., 2017; Agbozo, Colecraft, Jahn, & Guetterman, 2018; Tekle et al., 2019).

Although promotion activities affirming the importance of GMP may encourage additional use, it is important to consider the opportunity cost associated with consistent utilization at health facilities. Our findings are consistent with other studies that found that routine GMP utilization was simply one of many responsibilities of beneficiaries and, as a result, the inaccessibility of the facility‐based service potentially superseded its known importance (George, Latham, Abel, Ethirajan, & Frongillo, 1993; Roesler, Smithers, Winichagoon, Wangpakapattanawong, & Moore, 2018). Rural mothers, who may be largely unable to access transportation, living in areas with poor infrastructure or overburdened with agricultural or household duties, may need to carry their children long distances—sometimes in monsoon rains, intense heat, through snow, and over hills and mountainous terrain—to reach the nearest health facility. Once there, many are met with long lines, crying children and limited or non‐existent seating (Garha, 2016; Overseas Development Institute, 2016; Roesler et al., 2018). Because GMP requires frequent use, proper monitoring and recording, and timely and appropriate nutrition counselling to produce clear results, the service must be readily available and accessible to ensure that frequent GMP use is feasible. As such, community‐ and home‐based interventions may offer a stark advantage over facility‐based options, particularly for beneficiaries identified as prone to inconsistent attendance (Agbozo et al., 2018; George et al., 1993; Arole, 1998; Mayhew, Ickx, Stanekzai, Mashal, & Newbrander, 2014).

On the supply side, our results indicate that deficits in frontline worker service‐specific knowledge and skills impact the quality of care received. Although all health workers have theoretically received updated training on the administration of GMP services, this study found that this is not the case in practice. Given that most frontline workers failed to correctly measure, record and interpret the child's growth status and very few had received training on counselling methods, it is unsurprising that the ‘promotion’ component of GMP services is particularly weak, both in Nepal and in other countries (Tekle et al., 2019; Melkamu, Bitew, Muhammad, & Hunegnaw, 2019; Roesler et al., 2018; Bilal, Moser, Blanco, Spigt, & Jan Dinant, 2014). However, the recurrent theme of positive interactions between mothers' and frontline workers, particularly FCHVs, is promising. Although studies of GMP in other LMICs have noted that mothers reported feeling shamed for their child's growth status or deterred by frontline workers' unprofessional behaviours or negative attitudes, mothers in our study detailed beneficial and supportive interactions (Agbozo et al., 2018; Tekle et al., 2019). This discrepancy may be attributable to the high degree of visibility and admiration of FCHVs in Nepal, particularly as their role has evolved and expanded over time (Kandel & Lamichhane, 2019; Khatri, Mishra, & Khanal, 2017). It is critical that any future iterations of GMP capitalize on this enabling dynamic, providing frontline workers with the support and training needed to deliver quality counselling.

This study provides insight into GMP implementation and utilization using quantitative and qualitative approaches and fills a gap in the literature by including both service provider and beneficiary perspectives. Although data on use ever were collected, data on recurrent GMP use would strengthen our understanding of the state of GMP service utilization in Nepal. Furthermore, although this study did not have data on the fourth component of GMP, this in‐depth exploration of the first three components of GMP is unprecedented and critical to our understanding of service implementation and utilization. The qualitative IDIs and FGDs, although not generalizable to all of Nepal, enable an understanding of the variation of factors across the country given our sampling across agroecological zones and from east to west. As reducing persistent malnutrition is a high priority, further research should investigate the use of FCHVs and other community‐based providers for strengthening GMP services in Nepal. Experimental implementation science studies at the district and subdistrict levels could also be done to test different implementation modalities for strengthening service provision for GMP and other health and nutrition services in various contexts.

Our findings show that future discussions regarding the merit of GMP in Nepal should focus on ways to increase both routine utilization and the consistent provision of quality GMP services for diverse communities across agroecological‐ and resource‐variable settings. Although GMP is largely ubiquitous in nature, it has become increasingly apparent that country‐level applications of the intervention differ substantially in purpose, design, implementation and effectiveness (Bégin et al., 2019). As Nepal continues to strive for significant reductions in child undernutrition, there is a compelling need for effective and equitable GMP implementation. This paper indicates that frontline workers responsible for GMP must be trained and reoriented en masse about its importance and correct implementation, particularly regarding promotive nutrition counselling. In addition to training, ongoing supportive supervision is vital to effective delivery of services at the community level. Further research is needed to evaluate the salience of current GMP protocols in the context of Nepal's evolving health system and to research the causal relationship between individual and contextual factors and service utilization.

## CONFLICTS OF INTEREST

The authors declare that they have no conflicts of interest.

## CONTRIBUTIONS

KC designed the study and conceptualized the manuscript, guided the analysis and supported in writing of multiple drafts. MMP conducted the analysis, conducted the literature review and drafted the manuscript. SM supported the design of the qualitative data collection tools and methodology and provided editorial support. KPL, RNM and VD provided extensive editorial support and knowledge of GMP implementation in Nepal. PP provided editorial support and knowledge of *Suaahara II* programmatic details. MMP provided support for the qualitative analysis and the writing of multiple drafts. All authors reviewed multiple drafts of the manuscript and read and approved the final version.
